# Mito-nuclear interactions as drivers of gene movement on and off the X-chromosome

**DOI:** 10.1186/1471-2164-15-330

**Published:** 2014-05-02

**Authors:** Björn Rogell, Rebecca Dean, Bernardo Lemos, Damian K Dowling

**Affiliations:** School of Biological Sciences, Monash University, Clayton, 3800 Australia; Molecular and Integrative Physiological Sciences Program, Department of Environmental Health, Harvard School of Public Health, Boston, MA 02115 USA

**Keywords:** Gene expression, Sexual selection, Sexual conflict, Genomic conflict, mtDNA, Genome evolution

## Abstract

**Background:**

Mito-nuclear gene interactions regulate energy conversion, and are fundamental to eukaryotes. Generally, mito-nuclear coadaptation would be most efficient if the interacting nuclear genes were X-linked, because this maximizes the probability of favorable mito-nuclear allelic combinations co-transmitting across generations. Thus, under a coadaptation (CA) hypothesis, nuclear genes essential for mitochondrial function might be under selection to relocate to the X-chromosome. However, maternal inheritance predisposes the mitochondrial DNA (mtDNA) to accumulate variation that, while male-harming, is benign to females. Numerous nuclear genes were recently reported in *Drosophila melanogaster*, which exhibit male-specific patterns of differential expression when placed alongside different mtDNA haplotypes, suggesting that nuclear genes are sensitive to an underlying male-specific mitochondrial mutation load. These genes are thus candidates for involvement in mito-nuclear interactions driven by sexual conflict (SC), and selection might have moved them off the X-chromosome to facilitate an optimal evolutionary counter-response, through males, to the presence of male-harming mtDNA mutations. Furthermore, the presence of male-harming mtDNA mutations could exert selection for modifiers on the Y-chromosome, thus placing these mito-sensitive nuclear genes at the center of an evolutionary tug-of-war between mitochondrion and Y-chromosome.

We test these hypotheses by examining the chromosomal distributions of three distinct sets of mitochondrial-interacting nuclear genes in *D. melanogaster*; the first is a list of genes with mitochondrial annotations by Gene Ontologies, the second is a list comprising the core evolutionary-conserved mitochondrial proteome, and the third is a list of genes involved in male-specific responses to maternally-inherited mitochondrial variation and which might be putative targets of Y-chromosomal regulation.

**Results:**

Genes with mitochondrial annotations and genes representing the mitochondrial proteome do not exhibit statistically-significant biases in chromosomal representation. However, genes exhibiting sex-specific sensitivity to mtDNA are under-represented on the X-chromosome, over-represented among genes known to be sensitive to Y-chromosomal variation, and among genes previously associated with male fitness, but under-represented among genes associated with direct sexual antagonism.

**Conclusions:**

Our results are consistent with the SC hypothesis, suggesting that mitochondrial mutational pressure selects for gene movement off-the-X, hence enabling mito-nuclear coadaptation to proceed along trajectories that result in optimized fitness in both sexes.

## Background

The proteins that comprise the mitochondrial respiratory chain, and thus drive oxidative phosphorylation (OXPHOS), are encoded by genes spanning two obligate genomes - nuclear and mitochondrial [1]. While the mitochondrial DNA (mtDNA) encodes only a small fraction of these genes, their products are nonetheless crucial [2]. Indeed, the products of mito-nuclear interactions are so vital to life, that traditionally it was thought that any genetic variation accumulating in the coding regions of the mtDNA should be deleterious to function and thus purified by selection, and that therefore the mitochondria should contain little genetic variation of evolutionary relevance [3, 4]. However, over the past two decades, a large body of empirical evidence has accumulated that shows that natural levels of mitochondrial allelic variance indeed contribute to phenotypic variation in the expression of evolutionarily important traits [5, 6], such as metabolism [7], life span and ageing [8–10], reproductive fitness [11, 12], viability [13, 14] and gene expression [15]. These findings have prompted a re-evaluation of the role of the mitochondrial genome in driving fundamental evolutionary processes [6, 16, 17], including the evolution of genome organization [18].

Epistatic interactions between mitochondrial and nuclear genes affect the expression of fitness-related traits, and selection is predicted to optimize high performing mitochondrial-nuclear (mito-nuclear) allelic combinations [6, 17, 19]. Results of studies, which reported hybrid breakdown upon the enforced disruption of coevolved mito-nuclear genomic combinations, support this contention [12, 20–22]. Nonetheless, the scope for realized co-adaptation between mitochondrial and nuclear genomes will be constrained by the genomic location of the nuclear genes that interact with the mitochondria, and hinge on differences in how mitochondrial and the nuclear alleles segregate across generations [18]. While the mitochondrial genome generally follows a mode of strict maternal inheritance, autosomal chromosomes are bi-parentally transmitted and will therefore co-segregate with the mtDNA in 50 per cent of cases; while in XY sex chromosomal systems, the X-chromosome will co-segregate with the mtDNA in two-thirds of cases simply because females harbor two copies of the X, whereas males harbor one. In contrast, the mtDNA never co-segregates with the male sex-chromosome - the Y. Thus, these simple rules of co-segregation dictate that epistatic selection for optimized mitochondrial function will be most efficient if the interacting nuclear genes are X-chromosome linked, thus increasing co-segregation of mito-nuclear allelic pairings that work well together, across generations. Accordingly, selection should historically have favored an evolutionary pathway that side-steps the constraints imposed by inheritance asymmetries between mitochondrially- and nuclear-encoded genes, and maximizes co-transmission of alleles involved in mito-nuclear interactions [18]. This is the coadapted (CA) mito-nuclear hypothesis, and it predicts that nuclear genes involved in mito-nuclear interactions that encode important mitochondrial functions, should be enriched on the X-chromosome.

In a recent test of the CA hypothesis, Drown et al. [18] conducted an analysis across 14 mammalian species with XY systems. The Drown et al. study addressed whether nuclear genes classified as belonging to the Gene Ontology “cell component: mitochondria” were over-represented on the X-chromosome. Their results ran counter to prediction – with the “mitochondrially annotated (***mito-annotated***)” genes under-represented on the X.

This spurred the authors to invoke an alternative hypothesis, based on sexual conflict (SC), to explain the patterns. The SC hypothesis centers on the evolutionary prediction that maternal inheritance of the mtDNA will impede the ability of selection to directly remove mutations from the mtDNA sequence when these mutations are male-biased in their phenotypic effects [15, 23–25]. Thus, adaptive trajectories of mtDNA sequence evolution will primarily be driven by direct selection on females, and this will favor the fixation of female-benign (or –friendly), but potentially male-harming mtDNA mutations. This hypothesized phenomenon has been termed ‘Mother’s Curse’ [24], and the evolutionary process that leads to it a ‘sex-specific selective sieve’ in mitochondrial genome evolution [15, 23]. If the existence of fixed male-harming mtDNA mutations was biologically realized in natural populations, and of evolutionary consequence, then these mutations should in turn place male-biased selection on the nuclear genome for counter-adaptations that mitigate the negative effects of the mutations [6, 12, 19, 23]. Hence, to maximize the male-driven counter-response, nuclear genes whose function is subject to interference by the presence of male-harming mtDNA mutations might have been relocated off the X-chromosome throughout the course of evolutionary history. Doing so, would not only reduce the capacity for directly sexually antagonistic trajectories of mito-nuclear coevolution, but increase the capacity for nuclear modifier alleles to evolve that mitigate the effects of male-harming mtDNA mutations that have reached fixation in populations. Thus, the placement of these ‘mitochondrially-sensitive (***mito-sensitive***)’ nuclear genes on the autosomes [18], and off the X, would drive compensatory mito-nuclear trajectories in a direction that optimizes fitness in both sexes.

A recent study provided insights into the Mother’s Curse hypothesis, and identified a list of candidate mito-sensitive genes that respond to the presence of putatively male-harming mtDNA mutations in *Drosophila melanogaster* [15]. The authors placed five divergent mtDNA haplotypes, each replicated, alongside an isogenic nuclear background devoid of segregating allelic variance, and observed that around 10% of all nuclear transcripts in males (1212 genes), but close to ~0% in females (7 genes), were sensitive to the mtDNA sequence, and differentially expressed across haplotypes. Approximately one-third of these mito-sensitive nuclear transcripts were highly male biased in expression, and largely limited in expression to the male reproductive tissues [15]. These results provided strong support for the idea that purifying selection has removed the phenotype-modifying allelic variance from the mitochondrial genome that affects female function, but has missed a class of mutations that are male-biased in their effects. Thus, the function of these mito-sensitive nuclear genes is presumably subjected to negative interference by a male-specific mitochondrial mutation load.

Here, we take a novel approach to testing the co-adaptation (CA) and sexual conflict (SC) hypotheses of mito-nuclear genome architecture, following on from the framework introduced by Drown et al. [18] and utilized by Dean et al. [26]. Unlike the comparative vertebrate framework of Drown et al. [18], we focus our attention on patterns of nuclear gene placement in *D. melanogaster*, and test the CA and SC hypotheses across three distinct datasets of candidate nuclear genes for mito-nuclear interactions. The first is the list of genes annotated with a mitochondrial function using Gene Ontology annotations in Biomart [27], for *D. melanogaster*. Such mito-annotated genes putatively serve important roles in ensuring mitochondrial function, and include those that encode the 73 polypeptide subunits involved in mitochondrial respiratory chain assembly. However, as the protein interactions between mitochondria and nucleus have hitherto been poorly characterized, the completeness and informativeness of this set remains unclear [28]. Nevertheless, the list is useful for allowing direct comparison with the paper of Drown et al. [18], who used these annotations as the basis of their inferences. The second list of genes comes from a recent study that combined functional assays and protein expression measurements across humans, mice and Drosophila, to redefine the core mitochondrial proteome, consisting of around 400 conserved proteins [28]. The nuclear genes (*core mito-proteome*) encoding these proteins are expected to be explicitly tied to critical co-adaptive mito-nuclear processes. While one would predict, under the CA hypothesis, that both of these sets of genes (*mito-annotated* and *core mito-proteome*) would evolve to be enriched on the X-chromosome, the data from *mito-annotated* genes across mammals suggest otherwise, and implies that male-harming mitochondrial mutation pressure has favored their movement off-the-X [18].

We start by testing whether the patterns reported for mito-annotated genes in mammals apply to the quintessential XY invertebrate model species, *Drosophila melanogaster*, and we then test whether the core mito-proteome genes exhibit chromosomal distribution biases in favor of the CA or SC hypothesis. We then focus our attention on the list of ‘*mito-sensitive*’ genes, which contains genes that are for the most part highly male-biased in their sensitivity to the mtDNA sequence, and putative recipients of negative interference attributable to cryptic male-harming mtDNA mutations. This list represents a new set of candidate genes for involvement in sex-specific, potentially sexually antagonistic, mito-nuclear coevolution, and selection might have acted, throughout evolutionary history, to place these genes off the X-chromosome.

We then test whether the mito-sensitive nuclear genes are over-represented among genes that exhibit differential expression across diverse Y-chromosomes [29]. By doing so, we explore the possibility that mtDNA-induced interference of these male expression-specific nuclear genes has provoked an evolutionary response in the paternally-transmitted Y-chromosome, to counter the negative effects of male-harming mtDNA mutations. Given that the mtDNA and Y-chromosome follow opposed modes of asymmetrical inheritance, there is no scope for inter-genomic co-adaptation via passage of favorable mito-Y allelic combinations across generations. However, Y-chromosomes are likely to be under selection for the evolution of modifier alleles that wrest control over expression patterns within the male transcriptome, and the core control of male fertility, back over to genes that are paternally inherited.

Finally, we probe the putative mechanistic underpinnings of the male-specific mitochondrial mutation loads, asking whether these loads consist predominantly of mutations that are largely benign (and accumulated under a model of mutation-selection balance and drift), or beneficial (and accumulated under sexually antagonistic selection), to female function. We do this by exploring whether the mito-sensitive nuclear genes are over-represented on lists of genes known to be associated with either male-specific, or dedicated sexually antagonistic, fitness outcomes in *D. melanogaster* [30].

## Material and methods

The list of mito-annotated genes for *D. melanogaster* was sourced from Biomart, and represent all genes that have a mitochondrial cellular component annotation, as specified by the Gene Ontology ID 0005739 (524 genes). The list of core mito-proteome genes was sourced directly from Lotz et al. [28], and includes 419 genes whose essential contribution to mitochondrial proteomic function is common across humans, mice and fruit flies. The list of mito-sensitive genes was derived from the Innocenti et al. [15] study. We re-analysed these gene expression data, whereby 1211 nuclear genes were differentially expressed when placed alongside five different mtDNA haplotypes of male *D. melanogaster*. In all tests described below, the properties of the genes included in the mito-annotated and core mito-proteome lists were compared to the properties of all annotated genes (15493 unique genes). The properties of the mito-sensitive genes were compared to the properties of all unique annotated genes for which probe-sets are present on the Affymetrix GeneChip Drosophila Genome 2.0 that was used in Innocenti et al. [15] (12150 unique genes in total). Our methods are analogous to the methods used by Drown et al. [18].

Following the rationale outlined in the Introduction, we tested if the *mito-annotated*, the *core mito-proteome*, and the *mito-sensitive* nuclear genes, each of which are candidates for involvement in mito-nuclear interactions, are over- or under-represented on the X-chromosome using Fisher's exact test. We continued by testing the representation of genes in the mito-sensitive list among a dataset of genes known to react to genetic variation harbored within the Y-chromosome. This dataset of Y-sensitive genes was obtained from Lemos et al. [29], who investigated patterns of differential expression within the male transcriptome, when a standardized nuclear background was expressed alongside four distinct Y-chromosomes derived from disjunct natural populations. In addition, in order to probe putative mechanistic explanations to the observed patterns, we tested whether the mito-sensitive genes exhibit biases in their representation amongst candidate genes for female-specific, male-specific, sexually-antagonistic and combined fitness. These gene lists were obtained from Innocenti and Morrow [30].

In 2010, Gallach et al. [31] reported a list of nuclear duplicated genes with mitochondrial annotations (n = 123 genes), and found, consistent with Drown et al’s [18] findings, that these genes show avoidance of the X chromosome when duplicating. These duplicates exhibited strong male biases in expression, similar to those observed among the list of mito-sensitive genes [15]. Therefore, we examined the possibility that any patterns of chromosomal bias among the mito-sensitive gene list are driven by biases among the 123 nuclear gene duplicates with mitochondrial annotation, by analyzing the dataset when including and excluding these 123 genes.

In order to probe putative mechanistic explanations to the observed patterns, we tested whether the mito-sensitive genes exhibit biases in their representation amongst candidate genes for female-specific, male-specific, sexually-antagonistic and combined fitness. These gene lists were obtained from Innocenti and Morrow [30].

Finally, differential expression across gonads and somatic tissues has previously been linked to genome organization [32, 33]. For example, in *Drosophila*, genes with expression patterns biased to the male gonads are known to be under-represented on the X-chromosome [32]. To further investigate whether our analyses of genome organization, for nuclear genes involved in mito-nuclear interactions, are driven foremost by genes that exhibit testes-biased expression, we replicated all analyses, using gene-lists in which we removed all genes that are significantly over-expressed in the testes [identified using Flyatlas [34], ~2500 genes in total representing 16% of the total number of annotated genes].

## Results

**Figure 1:**
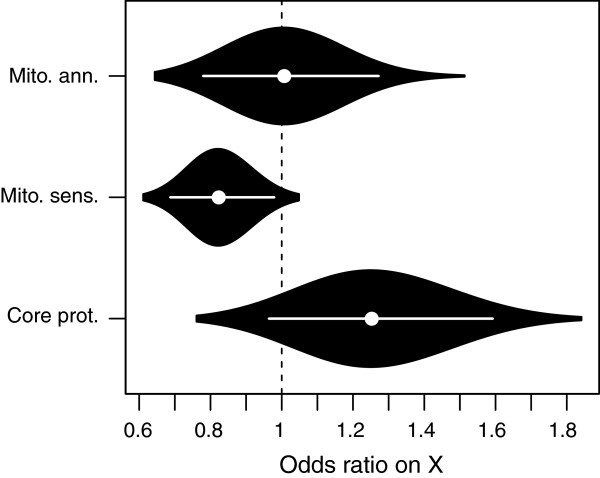
**Violin plot of the bootstrapped densities of odds ratios of X chromosomal placement vs. placement on other genomic regions across three groups of genes.** Genes annotated with a mitochondrial function (“Mit. ann.”), mito-sensitive genes in terms of differential gene expression (“Mit. sens”), and genes encoding mitochondrial core proteins (“Core prot.”). Dots indicate the bootstrapped median, and error bars the associated 95% confidence intervals.

There is substantial overlap between the identity of genes comprising the *core mito-proteome* and *mito-annotated* gene lists (overlap, 58%, P < 0.001). The list of mito-sensitive genes was however largely distinct from each of the other two lists (overlap *mito-sensitive* and *mito-annotated*: 9.7%, P = 0.15, *mito-sensitive* and *core mito-proteome*: 9%, P = 0.4). We found no bias amongst *mito-annotated* genes towards over- or under-representation on the X-chromosome (mito-annotated on X: 15.2%, general representation on X among all annotated genes: 15.5%, odds ratio: 1.01, P = 0.95, Figure 1). However, there was a trend toward higher representation of *core mito-proteome genes* on the X-chromosome (mito-protein genes on X: 18.7%, general representation on X among all annotated genes: 15.5%, odds ratio: 1.26, P = 0.078, Figure 1). This was however, not statistically significant, and therefore we detected no support for either the CA or SC hypotheses amongst genes encoding core mitochondrial proteins.

Consistent with our predictions based on the SC hypothesis, the *mito-sensitive* genes are under-represented on the X-chromosome, compared to the complete set of genes present on the Affymetrix chip (mito-sensitive genes on X: 13.7%, general representation on X among all genes on the Affymetrix chip: 16%, odds ratio: 0.82, P = 0.02, Figure 1). This effect remained after removal of the gene duplicates identified by Gallach et al. [31] (mito-sensitive genes on X: 13.4%, general representation on X among all genes on the Affymetrix chip: 16%, odds ratio: 0.8, P = 0.01). These *mito-sensitive* genes are also over-represented on a list of genes known to be sensitive to Y chromosomal regulation (*mito-sensitive* among Y-sensitive: 7.5%, general representation of Y-sensitive among all genes on the Affymetrix chip: 5.8%, odds ratio: 1.38, P = 0.008).

Furthermore, the *mito-sensitive* genes are under-represented on lists of genes associated with female-specific (female fitness genes among mito-sensitive genes: 2.8%, general representation of female fitness genes among all genes on the Affymetrix chip: 4.1%, odds ratio: 0.67, P = 0.02) and sex-general fitness (fitness genes among mito-sensitive genes: 2.6%, general representation among all genes on the Affymetrix chip: 3.9%, odds ratio: 0.64, P = 0.01) outcomes. There is also an under-representation of these *mito-sensitive* genes amongst a list of genes that have been previously associated with sexually antagonistic fitness outcomes (antagonistic fitness genes among mito-sensitive genes: 8.1%, general representation among all genes on the Affymetrix chip: 9.7%, odds ratio: 0.83, P = 0.046). However, the *mito-sensitive* genes are over-represented on a list of genes associated with male fitness (male specific fitness genes among mito-sensitive genes: 8.3%, general representation among all genes on the Affymetrix chip: 5.9%, odds ratio: 1.52, P <0.001).

Finally, we note that 16.1% of all annotated genes are over-expressed in the testes, and these testes-biased genes are over-represented amongst the *mito-annotated* genes (19%, odds ratio: 1.27, P = 0.03), *mito-sensitive* genes (41%, odds ratio: 3.3, P < 0.001) and Y-sensitive genes (25%, odds ratio: 1.8, P < 0.001), but under-represented among the *core mito-proteome* genes (7%, odds ratio: 0.39, P < 0.001). When re-analyzing the results presented above, following the removal of all genes exhibiting over-expression in the testes, the pattern for movement off-the-X-chromosome for *mito-sensitive* genes disappeared (mito-sensitive genes on X: 15.9%, general representation on X among all genes on the Affymetrix chip: 16.8%, odds ratio: 0.94, P = 0.58). Moreover, after removing these testes-biased genes, these *mito-sensitive* genes were also randomly-represented on a list of genes known to be sensitive to Y chromosomal regulation (*mito-sensitive* among Y-sensitive: 6.2%, general representation of Y-sensitive among all genes on the Affymetrix chip: 5.2%, odds ratio: 1.15, P = 0.39).

When analyzing our results excluding all testis-biased genes, there were still no biases amongst *mito-annotated* genes towards over- or under-representation on the X chromosome (mito-annotated on X: 16.7%, general representation on X among all annotated genes: 16%, odds ratio: 1.01, P = 0.46). However, the trend toward higher representation of *core mito-proteome genes* on the X-chromosome remains (mito-protein genes on X: 19.3%, general representation on X among all annotated genes: 16%, odds ratio: 1.26, P = 0.08).

## Discussion

Chromosomal placement of the nuclear genes embroiled in mito-nuclear interactions should be biased towards the X-chromosome in the case of co-adaptation, or away from the X-chromosome in the case of male-biased mitochondrial mutational pressure. Here, we harnessed three lists of candidate nuclear genes for mito-nuclear interactions to uncover patterns of chromosomal distribution. The first was a list of genes comprised of core mitochondrial cellular annotations, which are hypothesized to evolve along tight co-adaptive trajectories with their mtDNA-encoded counterparts [6, 18]. The second list comprised genes that make up the core and evolutionarily conserved mitochondrial proteome [28]. The third list comprised genes that are highly male-biased in expression and whose expression, in males but not females, is sensitive to allelic variation harbored within the mitochondrial genome [15].

The CA hypothesis predicts that placement of the genes, in both of the *mito-annotated* and *core mito-proteome* lists, onto the X-chromosome would augment the capacity for tight mito-nuclear co-adaptation. Yet, a recent study on mammals, using genes with mitochondrial annotation only, reported results that suggested that sexually antagonistic mitochondrial mutational pressure might have selected for gene movement off-the-X [18], with the benefits of doing so outweighing the costs of dampening the efficiency of mito-nuclear co-adaptation.

Contrary to predictions of either CA or SC, the mito-annotated genes of *D. melanogaster* do not exhibit biases for over- or under-representation on the X-chromosome. Furthermore, the genes that represent the core mitochondrial proteome exhibit no definitive pattern of bias in their chromosomal distribution, but if anything appear to follow a trend of over-representation on the X, in line with the CA hypothesis.

The *mito-sensitive* list comprises genes that are highly male-biased in expression, and whose expression, in males but not females, is sensitive to allelic variation harbored within the mitochondrial genome [15]. These genes are therefore overt candidates to be reacting to an underlying male-specific mitochondrial mutation load, and evolve along sex-specific and compensatory mito-nuclear trajectories [6, 12, 17, 19]. We therefore predicted these genes would be under-represented on the X, and also at the heart of an inter-sexual tug-of-war between maternally inherited mtDNA and paternally inherited Y-chromosomes. Consistent with these predictions of SC, we indeed found that these *mito-sensitive* genes are both under-represented on the X-chromosome, and over-represented on a list of genes whose expression has been shown to be sensitive to Y chromosomal regulation. Moreover, as predicted, these *mito-sensitive* genes are enriched for gene functions that are explicitly tied to male, but not female, fitness outcomes, with under-representation on a list of identified genes embroiled in sexually antagonistic fitness outcomes [30]. These patterns imply that neutral mutation accumulation of male-harming mtDNA mutations, rather than direct sexual antagonism *per se*, might provide the explanatory mechanism underlying the male-specific expression interference observed across mitochondrial haplotypes in Innocenti et al. [15]. We elaborate below.

Our findings follow on from the analysis by Drown et al. [18], which involved 16 vertebrate species (14 mammals exhibiting male heterogamety, and two birds exhibiting female heterogamety). The authors hypothesized that tight selection for mito-nuclear co-adaptation would favor the placement of the interacting nuclear genes onto the X-chromosomes of mammals (under the CA hypothesis), but would not favor placement of such genes onto the large sex-chromosome - the Z - in birds. These taxon-specific predictions were based on differences across XY and ZW systems in co-transmission rates of mtDNA with the larger, non-degenerate, sex-chromosome. Male heterogamety (via the XY system) results in increased co-transmission of mito-X relative to mito-autosomal allelic combinations, whereas female heterogamety (and the ZW system) results in decreased co-transmission of mito-Z combinations. Interestingly, and as something of an aside, these predictions overlook the possibility for tight mito-W co-adaptation in birds birds [but see 26 for *Schistosoma* example], given that alleles in the mtDNA and W-chromosome will co-transmit, through females, in 100% of cases. On the one hand, this omission seems reasonable given that the scope for adaptive movement of genes onto the W (and Y-chromosome) might be curtailed given this chromosome lacks recombination [35]. On the other hand, new evidence has emerged for adaptive evolutionary responses to female-specific selection on the W chromosome [36], and in this regard it is worth noting that any movement of mito-related nuclear genes onto the W will presumably augment the capacity for sexually antagonistic trajectories of mito-nuclear coevolution, with mito-W gene complexes optimized for female function, at the expense of male function.

Drown et al. [18] found general under-representation of *mito-annotated* genes on the X in mammals, with no biases in pattern in birds. Given the direction of the result in mammals ran counter to that predicted under CA, they formulated the SC hypothesis of *mito-annotated* gene placement to explain the results. On the one hand, our results using mito-annotations of *D. melanogaster*, were not concordant with the results gained from the same GO term search in mammals, which may bring into question the general validity of the SC hypothesis as a driver of gene movements for mito-interacting nuclear genes, particularly among XY systems [26]. Indeed, when examining the genomic location of genes tied to the core and conserved mitochondrial proteome, there was a non-significant trend in the opposite direction to that found by Drown et al. [18], i.e. over-representation on the X. On the other hand, we were able to explicitly test the SC hypothesis by using a novel list of genes, in *D. melanogaster*, that have been shown to be sensitive to mtDNA polymorphisms in males, but insensitive to these same polymorphisms in females. These *mito-sensitive* genes are therefore clear candidates to be under perpetual selection to move off the X-chromosome, under SC, and our results supported this.

Our study also provides insight into a possible evolutionary struggle for dual control over the function of the male transcriptome. The mitochondrial genome and the Y-chromosome share several similarities – both are diminutive in size and number of protein-coding genes, non-recombining, and unisexually-transmitted across generations [6, 37, 38] – the mitochondrial along the maternal, and the Y along the paternal lineage. Both were traditionally considered to be passive bystanders in adaptive evolutionary processes. Yet, results of studies investigating the functional significance of polymorphisms in mitochondrial DNA, and those in Y-chromosomes, suggest that these two genomic regions routinely harbor cryptic polymorphisms that have manifold effects on both genome-wide expression profiles and sex-specific fitness outcomes [12, 15, 29, 39, 40]. As such, the mitochondrion and Y-chromosome are candidates to accumulate sexually antagonistic adaptive polymorphisms (via intra- or inter-locus sexually antagonistic selection on the mtDNA, or inter-locus sexually antagonistic selection on the Y) that bolster the fitness of the transmitting sex, but that come at the expense of fitness in the non-transmitting sex. Our results are partially concordant with this idea. Specifically, we have shown that the nuclear genes that are sensitive to cryptic mtDNA polymorphism, and whose expression is modified in males but not in females, overlap significantly with those genes that are sensitive to cryptic Y-chromosome polymorphisms in males. This raises the intriguing analogy of the male transcriptome operating as an orchestra with two conductors – each with its own view on the trajectory of the ensemble. Yet, lack of recombination, together with low effective population sizes, of both mitochondrial genome and Y-chromosome suggest non-adaptive processes will play a considerable role in the accumulation of phenotype-changing polymorphisms on each gene region. Currently, it is unclear whether male-specific mitochondrial mutation loads are primarily underpinned by sexually antagonistic polymorphisms under positive selection in females [15, 25], or polymorphisms that are neutral or near-neutral in females and accumulating under drift [23, 24]. Insights from our study, however, point to the latter, because we found that the list of *mito-sensitive* genes was enriched for candidate genes that are associated with male-specific fitness, but under-represented by genes that have previously been associated with dedicated sexually-antagonistic fitness outcomes.

Gene movement can occur via two sets of processes – those at the DNA level (recombination- or transposable element-driven duplication), or at the RNA level (retroposition via the reverse transcription of mRNA - retrogenes). An excess of DNA-based and RNA-based relocations out of the X-chromosome has been reported in *Drosophila*. Most retrogenes in *D. melanogaster* that moved off the X, are over-expressed in the testes, and this pattern has been attributable to selection for testes-biased genes on the autosomes in order to avoid spermatogenesis X-inactivation [32, 33], as well as to generally circumvent the scope for sexual antagonism [31, 32] in light of the knowledge that the X-chromosome is a hotspot for sexually antagonistic fitness variation [41, 42]. When we removed genes that exhibit significant over-expression in the testes from our analyses, the pattern among the mito-sensitive genes for under-representation on the X-chromosome disappeared. This is, however, not surprising, considering that the *mito-sensitive* genes are, by definition, those that are male-biased in their sensitivity to the mtDNA sequence (i.e. only significantly differentially expressed in males, across distinct mtDNA haplotypes [15]), and when reconciled with the knowledge that evolutionary theory predicts that it is male homologs of sexually dimorphic phenotypes that will be most susceptible to mitochondrial mutational pressure invoked by Mother’s Curse [15, 23, 43]. Indeed, the reproductive tissues such as the testes are prime candidates to succumb to this process, given their sex-limitation in expression [15]. It is acknowledged that multiple forces are likely acting to move genes with testes-biased expression away from the X-chromosome [31, 32]. We contend that our results, together with those of Drown et al. [18], invoke a new twist on this long established pattern, by indicating that perpetual accumulation of sex-specific mitochondrial mutations constitutes a hitherto unrealized driver of this out-of-X movement for testes-specific genes. This is further supported by Gallach et al. [31], who examined a list of 512 nuclear encoded mitochondrial genes in *Drosophila*, and identified 123 genes involved in gene duplication. They reported that the majority of these duplications involved relocation of the duplicate away from the parental gene, with relocations off the X-chromosome to the autosomes being 60% more frequent than expected by chance. Conversely, relocations from autosomes to X-chromosome were over 70 per cent less frequent than expected by chance. Furthermore, most (83%) of the relocated nuclear encoded mitochondrial gene duplicates exhibit testes-specific expression. The concordance between patterns involving the 123 gene duplicates in that study, and the 1211 plus *mito-sensitive* nuclear genes in this study, both in terms of chromosomal distribution away from the X, and in terms of tendency for testes-specific expression, is strong; and supports the idea that male-specific mitochondrial mutation loads might underpin these patterns.

We also observed interesting patterns of chromosomal representation for testes-biased genes on the *mito-annotated* and *core mito-proteome* data sets. The list of mito-annotated genes contains an over-representation of genes exhibiting testes-biased expression. In contrast, the list of core mito-proteome genes actually contains a paucity of testes-biased genes. This reconciles the notion that the fundamental basal genetic machinery on which mitochondrial integrity hinges, should not involve genes that exhibit sex-specific function and promote selection for core mitochondrial optimization in one sex at the expense of the other. Core mitochondrial functionality is key to eukaryote life, and thus there should be strong selection for the most salient of genetic machinery involved in mitochondrial processes to serve the best interests of each sex equally.

## Conclusions

In conclusion, recent theoretical and empirical advances on the dynamics of mitochondrial genome evolution show that mitochondrial genetic variation is associated with male-biased effects on the expression of core life-history traits [12, 15, 22, 39]. By examining the chromosomal distribution of nuclear genes that are differentially expressed across mitochondrial haplotypes in male, but not female, *Drosophila melanogaster*, we found patterns that are consistent with, and extend on, results of previous studies that analyzed the chromosomal distribution of nuclear genes annotated for mitochondrial function [18, 31]. These genes are under-represented on the X-chromosome, consistent with the SC hypothesis. However, the genes tend to be under-represented among candidate genes for sexually-antagonistic fitness, and are over-represented among candidate genes for male-specific fitness. This implies that neutral mutation-accumulation, rather than sexually-antagonistic selection, in mitochondrial genomes might well underpin the male-specific fitness-losses associated with mitochondrial mutation loads in *D. melanogaster*.

### Availability of supporting data

Microarray data for Innocenti et al. [15] and Lemos et al. [29] is available on the Gene Expression Omnibus database, accession no. GSE24729 and GSE9457, respectively. Fitness data from Innocenti and Morrow [30] is available as supplementary information to their paper. Raw data from Lotz et al. [28] is available via their paper at http://pubs.acs.org/doi/abs/10.1021/pr400539j.
